# Highly Stable Liposomes Based on Tetraether Lipids as a Promising and Versatile Drug Delivery System

**DOI:** 10.3390/ma15196995

**Published:** 2022-10-09

**Authors:** Aybike Hemetsberger, Eduard Preis, Konrad Engelhardt, Bernd Gutberlet, Frank Runkel, Udo Bakowsky

**Affiliations:** 1 Department of Pharmaceutics and Biopharmaceutics, University of Marburg, Robert-Koch-Str. 4, 35037 Marburg, Germany; 2 Institute of Bioprocess Engineering and Pharmaceutical Technology, University of Applied Sciences Mittelhessen, Wiesenstrasse 14, 35390 Giessen, Germany; 3 Faculty of Biology and Chemistry, Justus-Liebig University Giessen, Heinrich-Buff-Ring 17, 35392 Giessen, Germany

**Keywords:** tetraether lipids, liposomes, stability, pulmonary drug delivery, oral drug delivery

## Abstract

Conventional liposomes often lack stability, limiting their applicability and usage apart from intravenous routes. Nevertheless, their advantages in drug encapsulation and physicochemical properties might be helpful in oral and pulmonary drug delivery. This study investigated the feasibility and stability of liposomes containing tetraether lipids (TEL) from *Thermoplasma acidophilum*. Liposomes composed of different molar ratios of TEL:Phospholipon 100H (Ph) were produced and exposed to various temperature and pH conditions. The effects on size, polydispersity index, and zeta potential were examined by dynamic and electrophoretic light scattering. Autoclaving, which was considered an additional process step after fabrication, could minimize contamination and prolong shelf life, and the stability after autoclaving was tested. Moreover, 5(6)-carboxyfluorescein leakage was measured after incubation in the presence of fetal calf serum (FCS) and lung surfactant (Alveofact). The incorporation of TEL into the liposomes significantly impacted the stability against low pH, higher temperatures, and even sterilization by autoclaving. The stability of liposomes containing TEL was confirmed by atomic force microscopy as images revealed similar sizes and morphology before and after incubation with FCS. It could be concluded that increasing the molar ratio in the TEL:Ph liposome formulations improved the structural stability against high temperature, low pH, sterilization via autoclaving, and the presence of FCS and lung surfactant.

## 1. Introduction

Liposomes are spherical lipid membranes consisting of one or more lipid layers. These lipid layers have hydrophilic and hydrophobic compartments [1,2]. Over the past decades, many advantages of conventional liposomes have been revealed, e.g., high versatility, good biocompatibility, and prolonged drug half-life, emphasizing their suitability as a tailor-made drug delivery system [3,4,5,6,7,8]. Biocompatible properties allow them to be used as delivery systems for drugs, proteins, plasmid DNAs, antisense oligonucleotides, or ribosomes, as well as for biochemical, pharmaceutical, and cosmetic purposes [9,10,11,12]. Besides the non-pathogenic and non-immunogenic features, their production is of relatively low cost. Therefore, liposomes seem suitable for different application routes, e.g., parenteral, transdermal, pulmonary, and oral drug delivery [13].

However, some drawbacks can be observed in the case of liposomes composed of distearoylphosphatidylcholine or dipalmitoylphosphatidylcholine, phosphatidylserine, and cholesterol. These drawbacks are low stability at low pH values, low stability against biological media, high fusion tendency with biological barriers, and rapid clearance by the reticuloendothelial system (RES) or fast metabolism [14]. Various studies were conducted and summarized in different review articles elucidating multiple approaches to enhance the stability of liposomes and their use in oral drug delivery [15,16,17].

**Figure 1:**
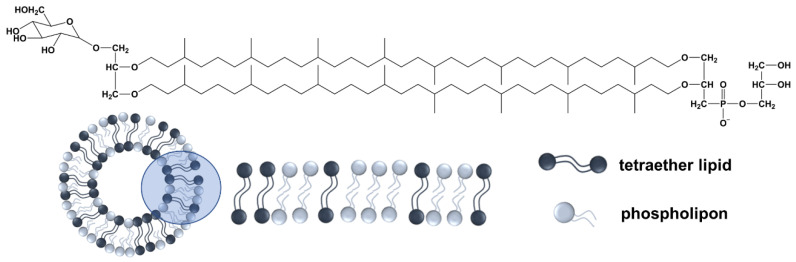
An illustration of the main structure of the isolated tetraether lipids and the membrane organization of the phospholipids (Phospholipon) and the TEL within the liposomes.

It is known that archaeal liposomes, which include saturated bipolar tetraether lipids (TEL) (Figure 1), are more stable than liposomes formed of bacterial or eukaryotic lipids [18,19,20]. Benvegnu et al. have also shown that adding synthetic TEL to conventional liposomes increases their stability [18]. Ozecetin et al. prepared liposomes containing TEL from thermophilic or hyperthermophilic archaea to overcome the drawbacks of conventional liposomes, i.e., *Thermoplasma acidophilum* or *Sulfolobus acidocaldarius*, and the impact on stability was investigated [21].

*Thermoplasma acidophilum* is a thermoacidophilic archaeon, first isolated in 1970 by Darland et al. [22]. The optimal living conditions for *Thermoplasma acidophilum* are at pH 2 and a temperature of 59 °C. The specific structure of lipids composing the cytoplasmic membrane of these archaea allows them to sustain extreme environmental conditions. The lipid constitution of archaeal membranes contains ether bonds and varying numbers of cyclopentane in the hydrocarbon chains providing a higher aqueous dispersity and an expanded bilayer membrane [18,19]. Furthermore, archaeal lipids do not contain double bonds. These properties distinguish archaea species like *Thermoplasma acidophilum* from bacterial species [23,24,25].

Liposomes prepared with archaeal lipids offer various medical and pharmaceutical applications because of their enhanced stability [26,27,28,29]. When incorporating a hydrophilic or lipophilic drug, liposomes become an attractive tool for reducing overall drug dose and decreasing side effects. Recently, oral peptide delivery using TEL liposomes gained more and more interest. Li et al. demonstrated that TEL liposomes were more stable in gastrointestinal fluids than conventional liposomes leading to longer retention times in vivo in BALB/c mice [30]. Continuous research on other peptides, e.g., octreotide, vancomycin, and Myrcludex, and their oral drug delivery emphasized the advantageous features of TEL liposomes [31,32,33]. According to Kimura et al., a significant quantity of drug-entrapped liposomes can be taken up by small-intestinal mucosa [34]. To reach these parts of the human gastrointestinal tract, a thorough investigation is necessary to establish liposomal formulations that demonstrate appropriate stability at different pH values and temperatures and in the presence of lipids and proteins [35,36]. Indeed, the essential criterion of a well-designed vesicle to be used in oral drug delivery is the ability to survive in the gastrointestinal tract environment. As a result of low pH values, most conventional liposomes undergo complete disintegration forming a mixture of soluble micelles [37,38].

On the other hand, the pulmonary system is also attractive for a liposomal drug delivery system. Liposomes smaller than 260 nm can be absorbed on the large surface of the alveolar epithelium. Particles larger than 260 nm are likely to be destroyed by the macrophage system of the lung [39]. Another criterion that affects stability in the pulmonary system is the influence of the natural surface surfactant of the lung. Hence, this study investigated liposome stability during exposure to the commercial lung surfactant Alveofact [40]. Pulmonary drug delivery also depends on the applied devices and the device parameters. It could be shown that the shear forces applied during nebulization are a crucial influencing parameter. Stabilization of a liposomal formulation during nebulization can also be achieved by tetraether lipids [41,42].

This study addresses the stability properties of TEL liposomes compared to phospholipid liposomes, providing a perspective on further usage of TEL liposomes as drug carriers in the gastrointestinal tract and pulmonary system.

## 2. Materials and Methods

### 2.1. Materials

A freeze-dried biomass of *Thermoplasma acidophilum* (TransMIT GmbH, Development of colloidal drug formulations, Giessen, Germany) was stored at −20 °C until usage. Phospholipon 100H (Ph; commercial phosphatidylcholine including stearic and palmitic acid) and 5(6)-carboxyfluorescein (CF) were purchased from Lipoid GmbH (Ludwigshafen, Germany) and Fluka, (Buchs, Switzerland), respectively. Fetal calf serum (FCS) was obtained from PAA (Coelbe, Germany), Triton X-100 was provided by Carl Roth (Karlsruhe, Germany). The lung surfactant Alveofact was provided by Lyomark Pharma (Oberhaching, Germany). Chloroform (CHCl_3_) and methanol (MeOH) were both purchased from Carl Roth (Karlsruhe, Germany) and the Chloroform:Methanol (CHCl_3_:MeOH) (2:1 *v*/*v*) mixture was stored at room temperature until it was used. HEPES buffer solution (HBS) was prepared with 40 mM HEPES (4-(2-hydroxyethyl)-1-piperazineethanesulfonic acid), 5 mM Glucose (Dextrose), and 120 mM NaCl and adjusted to pH 7.4. Phosphate-buffered saline (pH 7.4) composed of 1.3 mM KH_2_PO_4_, 7.4 mM Na_2_HPO_4_ × 2 H_2_O, 129 mM NaCl, and 2.5 mM KCl, which were dissolved in bidistilled water. All chemicals and solvents used in the experiments were of analytical grade.

### 2.2. Extraction of the Tetraether Lipids

TEL were extracted from 10 g freeze-dried biomass of *Thermoplasma acidophilum* according to a modified method of Bode et al. [43]. The biomass was transferred into a 1 L flask and mixed with 500 mL CHCl_3_:MeOH (2:1 *v*/*v*). The mixture was heated to a reflux temperature of 60 °C for 12 h. After cooling down to room temperature, the mixture was filtered through 300 g silica 60 (Merck, Germany). The filter cake was transferred to the flask and refluxed for another 12 h. After filtration, the solvent from the resultant mixture was evaporated under vacuum to yield a lipid residue. This residue consists of the total lipid fraction. From this total lipid fraction, 10 mg/mL TEL solution in CHCl_3_:MeOH (2:1 *v*/*v*) was prepared.

### 2.3. Preparation of Liposomes

Ph (100% saturated phosphatidylcholine) was dissolved in CHCl_3_:MeOH (2:1 *v*/*v*) to prepare a 10 mg/mL stock solution. Liposome suspensions were prepared in molar ratios of 10:0, 7:3, 5:5, and 0:10 TEL:Ph. These suspensions were transferred to 10 mL round bottom flasks and evaporated at 45 °C 300 mbar with a rotary evaporator (Heidolph, Laborota 400, Schwabach, Germany) to obtain a lipid film. First, the thin film was hydrated with bidistilled water containing 0.9% NaCl. The solution was sonicated with a probe-type sonicator with an increased energy input (G. Heinemann Ultraschall und Labortechnik Schwöbosch, Germany), and a homogeneous suspension of the liposomes in water was obtained. Sonication was applied for 8 min (30 s sonication followed by 30 s rest). After sonication, liposomes were filtered with syringe filters through a pore size of 0.2 µm. Finally, all the liposome suspensions were extruded 21 times through a 0.1 µm polycarbonate membrane filter by a mini extruder device (Avanti, Hamburg, Germany).

### 2.4. Preparation of 5(6)-Carboxyfluorescein-Loaded Liposomes

CF liposomes were also prepared according to the solvent evaporation method. The related lipid compositions with the same molar ratios were dissolved in CHCl_3_:MeOH (2:1 *v*/*v*) and evaporated at 45 °C and 300 mbar with a rotary evaporator. The lipid layers were hydrated with phosphate-buffered saline (pH 7.4), which contained 50 mM CF to form CF-loaded liposomes. After hydration, the same procedure was applied as mentioned above. The encapsulation efficiency (EE) was calculated according to the following equation:

(1) $$Encapsulation\;efficiency\;\left[\%\right]=\frac{c_{total}-c_{out}}{c_{total}}\times 100\%$$

where *C_out_* defines the fluorescence intensity not encapsulated in liposomes, and *C_total_* is the corresponding value after adding Triton X-100 to disturb the liposomes to find out the total CF concentration. Additionally, % CF leakage was also determined from the equation described by Ishii et al. [44]. These calculations were applied after incubation with Alveofact and FCS, respectively.

### 2.5. Vesicle Size and Zeta Potential Determination

Mean diameters and polydispersity indices (PDI) of liposomes were determined by dynamic light scattering (DLS) using a ZetaSizer (Nano Series; Malvern instruments GmbH, Kassel, Germany), with a scattering angle of 90° at a temperature of 25 °C. Before all the measurements, 10 µL liposome suspensions were diluted with bidistilled water in the ratio of 1:6 (*v*/*v*). The zeta potentials of the particles were determined by electrophoretic light scattering using a ZetaSizer (Nano Series; Malvern instruments GmbH, Kassel, Germany), and the measurements were performed in a folded electrophoresis cell with a 633 nm He-Ne Laser, scattering angle of 173°, at 25 °C. 70 µL of liposome suspension were diluted with 730 µL bidistilled water and measured in triplicates.

### 2.6. Atomic Force Microscopy

Morphology of the liposomes was observed using atomic force microscopy (AFM), which was performed on a JPK NanoWizard™ (Berlin, Germany) and a Digital Nanoscope IV Bioscope (Veeco Instruments, Santa Barbara, CA, USA). Additionally, the stability against FCS and Alveofact was determined by visualizing the liposomes before and after incubation with the respective medium. The AFM was tuned to provide acoustic and vibrational damping. Measurements were performed in tapping mode (intermitted contact), in which the cantilever oscillated with determined amplitude close to its resonance frequency, with scan rates from 0.5 to 1 Hz. A HQ:NSC16/AL_BS (Anfatec Instruments AG, Oelsnitz, Germany) cantilever was used. Data were processed using JPKSPM data processing software (v. 5.1.8, JPK instruments) [45].

### 2.7. Thermostability

Each liposomal formulation was diluted with bidistilled water containing 0.9% NaCl (1:6 *v*/*v*) and incubated for 4 h at 25 °C, 36 °C, and 60 °C. After incubation, the samples were cooled down to room temperature, and the mean diameters and PDI of liposomes were determined using DLS (2.5).

### 2.8. Stability during Autoclavation

The samples were autoclaved to evaluate sterilization potential. Autoclavation was performed on a standard autoclave 3850 ELC (Systec GmbH, Wettenberg, Germany). Samples were diluted with bidistilled water containing 0.9% NaCl (1:6 *v*/*v*) and were treated with a standard autoclavation procedure for solutions (15 min at 121 °C, 29 psi pressure, and saturated steam). After cooling down, the mean diameters and PDI of liposomes were determined using DLS (2.5).

### 2.9. pH Stability

The measurements were done with different pH values mimicking the gastrointestinal environment, and the effect of different acidity levels on the mean diameter of the liposomes was observed. 100 µL aliquots of liposome formulations were incubated in 600 µL of various pH buffer solutions; pH 2.0 (50 mL of 0.2 m KCl, 13 mL 0.2 m HCl), pH 4.0 (100 mL 0.1 m KHC_8_H_4_O_4_, 0.2 mL 0.1 m HCl), pH 7.4 (100 mL 0.1 m KH_2_PO_4_, 78.2 mL 0.1 m NaOH), pH 9.0 (100 mL 0.1 m C_4_H_11_NO_3_ and 11.4 mL 0.1 m HCl). After 4 days of incubation, the mean diameters and PDI were determined.

### 2.10. Stability in Lung Surfactant

The structural integrity of liposomes was measured after incubation with Alveofact, mimicking the conditions in the human respiratory system. 1 mL of total lipid solution was treated with 60 mg of Alveofact vial and 1 mL HBS mixture [46]. CF liposome solutions containing 50 mM CF were incubated in Alveofact solution with a ratio of 1:9 (*v*/*v*) [40]. To determine the CF leakage due to liposome disruption induced by Alveofact incubation, the samples were placed into 96-well plates, fluorescence measurements were performed using an excitation wavelength of 480 nm, and emission was detected at 530 nm. The system was set for the liposomal amounts to give a total fluorescence between 3000 and 6500 arbitrary fluorescence units (FU) at a gain of 55 FU to avoid quenching effects (Tecan Saphire 2, Tecan, Austria). The initial fluorescence was determined right after starting the incubation. To determine the total fluorescence, after 4 h of incubation at 37 °C, Triton X-100 (1.0% *v*/*v* concentration) was added to the CF liposomes in a volume ratio of 1:20 to disrupt CF liposomes [47].

### 2.11. Stability against FCS

Stability after FCS incubation was observed using commercial FCS. % *CF Leakage* was investigated for each one of the liposome formulations. The liposome formulations were diluted with FCS in a ratio of 1:2.5 (*v*/*v*) and incubated at 37 °C for 4 h. Right after starting incubation, the initial values were measured. The same fluorescence settings were used as explained above. The % *CF Leakage* was calculated according to the following equation:

(2) $$CF\;Leakage\;\left[\%\right]=\frac{F-F_{i}}{F_{f}-F_{i}}\times 100\%$$

where *F_i_* denotes initial fluorescence intensity, *F* is the intensity after 4 h incubation in related mediums and *F_f_* is the final intensity in the absence of Triton X-100 [48]. Leakage after Alveofact incubation was calculated accordingly.

### 2.12. Statistical Analysis

All experiments were performed in triplicates and the results are presented as mean ± standard deviation (S.D.) unless explicitly stated otherwise.

## 3. Results

### 3.1. Physicochemical Characterization

Liposomes were prepared with the compositions of 0:10, 5:5, 7:3, 0:10 TEL:Ph (molar ratio). The hydrodynamic diameter, zeta potential, and PDI were compared according to the TEL proportion. This data is presented in Table 1. To prepare unilamellar liposomes, the liposome solutions were extruded 21 times through a 100 nm extrusion filter [49].

The initial diameters of the liposomes vary from 34.56 nm to 135.60 nm. The PDI values were between 0.12 and 0.24, and all the liposomes were negatively charged. This negative charge increases with ascending TEL content. The potentials vary from −19.40 to −49.90 mV. With the increase of TEL proportion, the unilamellar liposome’s size also increases. The initial mean diameter, PDI, and zeta potential of the different formulations of the liposomes are listed in Table 1. The EE of the formulations was measured as explained in Section 2.4. The results were 9.4 mM (18.8 ± 3.81%) for 0:10 TEL:Ph, 11.36 mM (22.73 ± 0.21%) for 5:5 TEL:Ph, 10.21 mM (20.43 ± 5.71%) for 7:3 TEL:Ph, 10.24 mM (20.48 ± 4.94%) for 10:0 TEL:Ph. Overall, the liposomal formulations containing TEL could be prepared with well-defined sizes and suitable zeta potential.

### 3.2. Thermostability of the Liposomes

**Figure 2:**
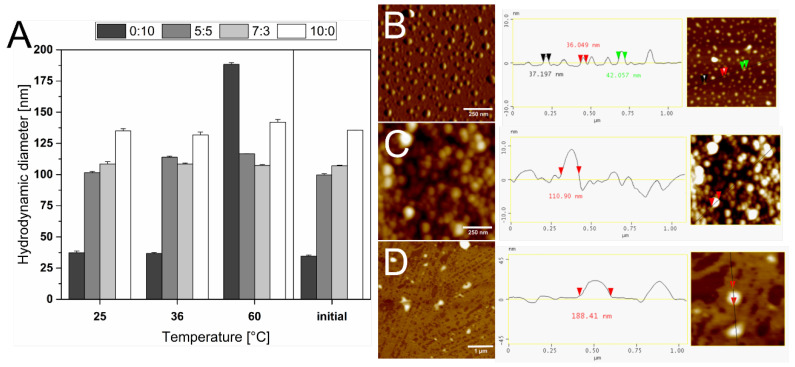
(**A**) The hydrodynamic diameter of liposomes composed of tetraether lipids (TEL) and Phospholipon 100H (Ph) in different molar ratios (0:10, 5:5, 7:3, 10:0; TEL:Ph) incubated for 4 h at 25, 36, and 60 °C compared to initial values. AFM images in height or phase mode of (**B**) 0:10 TEL:Ph liposomes incubated for 4 h at 25 °C, (**C**) 7:3 TEL:Ph liposomes incubated for 4 h at 25 °C, and (**D**) 0:10 TEL:Ph liposomes incubated for 4 h at 60 °C. Scale bars in B+C represent 250 nm. The scale bar in D represents 1 µm.

The relative thermostability of the liposomes was investigated by measuring the hydrodynamic diameter and PDI after 4 h of incubation at various temperature conditions. At room temperature and 36 °C, no significant difference was observed. However, at 60 °C, the size and PDI of the formulation 0:10 TEL:Ph increased from 34.56 ± 0.77 nm to 188.30 ± 1.39 nm and from 0.24 ± 0.01 to 0.44 ± 0.11, respectively. The other formulations containing TEL demonstrated better stability at higher temperature conditions. Hydrodynamic diameters are illustrated in Figure 2, and the PDI results after incubation are listed in Table 2.

It is reported that the permeability of TEL liposomes is less sensitive to temperature changes [50]. In our study, the mean diameters of all liposome formulations were measured and found to be below 0.2 µm and TEL fraction exhibits a large negative zeta potential in agreement with Komatsu et al. [51]. As the temperature increases to 60 °C, the size of liposomes formed with pure Ph increases too. These results show that TEL liposomes are less temperature-sensitive than phospholipon containing liposomes, which was reported to be the result of membrane permeability and viscosity properties of the extracted membrane lipids [52]. The PDI values for each composition under different temperatures indicate similar stability behavior except for the liposomes consisting of pure Ph.

### 3.3. Stability during Autoclavation

**Figure 3:**
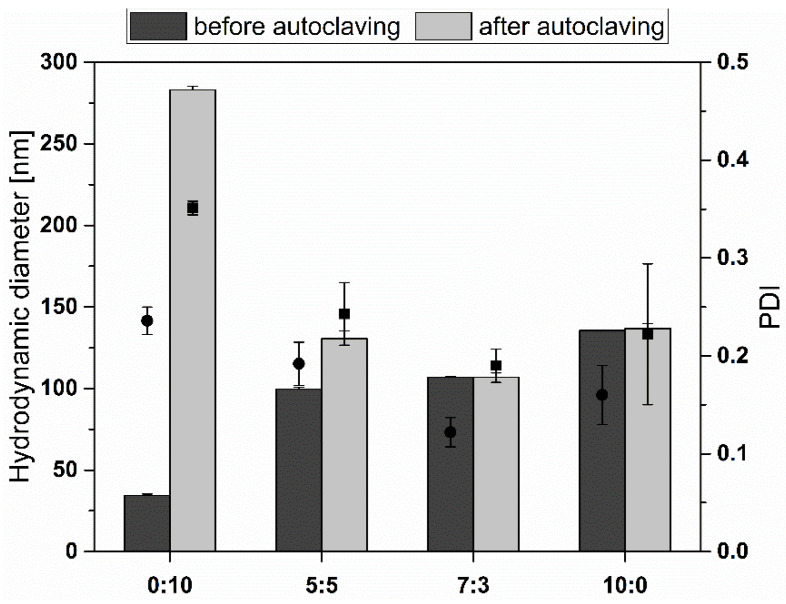
The hydrodynamic diameter and polydispersity index (PDI) of liposomes composed of tetraether lipids (TEL) and Phospholipon 100H (Ph) in different molar ratios (0:10, 5:5, 7:3, 10:0; TEL:Ph) before (filled circle) and after (filled square) autoclaving.

Clinical applications of liposomal vesicles require absolute sterility and pyrogen-free conditions. Since conventional lipids mostly degrade during heating or radiation, structural improvements are necessary to overcome these drawbacks [53,54]. Alternatively, 0.2 µm bacterial filters might be used to obtain sufficient sterility. However, their use is limited by the formulation size since this technique is only suitable for liposomes with a mean diameter smaller than 300 nm [55]. This proves to be a severe drawback for the clinical use of liposomes. Therefore, the stability of TEL liposomes was investigated under autoclavation conditions, according to Bode et al. [43]. Previously, we reported the autoclavation data for Glycerol Dialkyl Nonitol Tetraether (GDNT) liposomes [21]. Due to the high stability of TEL, we investigated the total polar lipids, and different TEL formulations were compared after autoclavation. Size and PDI values were determined (Figure 3). The liposome solutions were autoclaved at 121 °C for 15 min. While the TEL-containing liposomes are hardly affected by the increased temperature, 0:10 TEL:Ph liposomes seem to enlarge drastically, which agrees with the AFM images in Figure 2D displaying large-scale fusions even at 60 °C. The very high PDI also indicates a very inhomogeneous sample distribution. 10:0 TEL:Ph liposome formulations were intact after autoclavation, indicating high stability even at this temperature. The PDI values were under 0.20, and no significant difference was observed between the hydrodynamic diameter of liposomes before and after autoclavation.

Additional to the sterilization step, a limulus amebocyte lysate test was applied to evaluate the presence or absence of pyrogens. Gel formation could only be observed in the positive control (Control Standard Endotoxin), whereas the 10:0 TEL:Ph liposomes showed similar properties as the pyrogen-free water (Figure S1).

### 3.4. pH Stability

**Figure 4:**
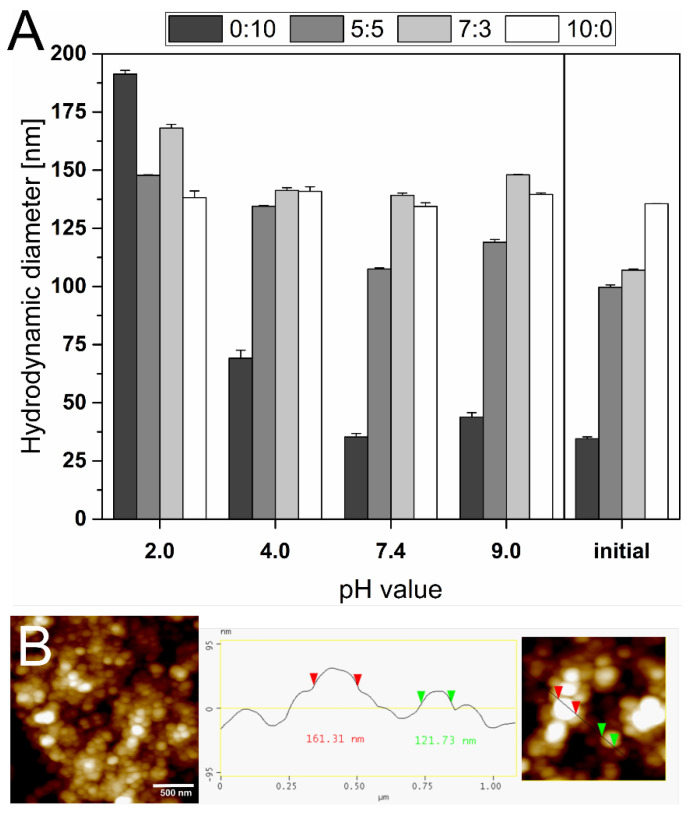
(**A**) The hydrodynamic diameter of liposomes composed of tetraether lipids (TEL) and Phospholipon 100H (Ph) in different molar ratios (0:10, 5:5, 7:3, 10:0; TEL:Ph) incubated for 4 d at pH 2.0, 4.0, 7.4, and 9.0, respectively, compared to initial values. AFM images in height or phase mode of (**B**) 7:3 TEL:Ph liposomes incubated for 4 d at pH 2.0. The scale bar in B represents 500 nm.

After incubation at different pH values, changes in hydrodynamic diameter and PDI of all liposomal formulations were monitored. As depicted in Figure 4, the hydrodynamic diameter of 0:10 TEL:Ph liposomes significantly increased more than 5 times its initial size at pH 2.0 (from 34.56 nm to 191.30 nm). Furthermore, the PDI of 0:10 TEL:Ph liposomes was nearly doubled at pH 2.0 (from 0.24 to 0.43), hinting at a multimodal distribution and a disruption of the lipid bilayer of the liposomes (Table 3). In his review article, Chong summarized that liposomes with more than 90% of bipolar TEL of total polar lipids extracted from *Thermoplasma* *acidophilum* are more stable than diester/diether lipid liposomes [19]. In contrast, in this study all liposomal formulations containing TEL showed more stability throughout the pH range from pH 2.0 to 9.0. Although the hydrodynamic diameter of liposomes composed of 5:5 and 7:3 TEL:Ph increased from 99.64 nm to 147.80 nm, and from 107.00 nm to 168.10 nm, respectively, only minor alterations of the PDI (<0.25) were observed. 10:0 TEL:Ph liposomes displayed negligible changes in hydrodynamic diameter (±5 nm) and PDI (±0.08) compared to the initial values in the pH range from pH 2.0 to pH 9.0. All liposomes with TEL still show a round shape when visualized with AFM, and no aggregations could be observed. In contrast, 0:10 TEL:Ph liposomes are not stable at low pH values, and large fusions are visible (Figure 4B). Elferink et al. reported that liposomes prepared from TEL mixtures offer much lower proton permeability [49]. This unique property is an adaptation of the archaea cell membrane to extreme environmental conditions to maintain the intracellular pH of the cell. The results shown in Figure 4 can be explained by the liposome membrane integrity at low pH, provided by the lipid backbone, the orientation of TEL, and bipolar head groups in their structures. Although *Thermoplasma* *acidophilum* grows optimally at pH 2, it has an internal pH of 6.9 [56,57]. To cope with this huge pH difference, *Thermoplasma* *acidophilum* has developed cytoplasmic membrane lipids, which consist of asymmetrical, negatively-charged phosphate headgroups [55,58]. Therefore, with increasing TEL component of the liposome formulation, proton permeability decreases, resulting in increased pH stability.

### 3.5. Stability against Alveofact and FCS

**Figure 5:**
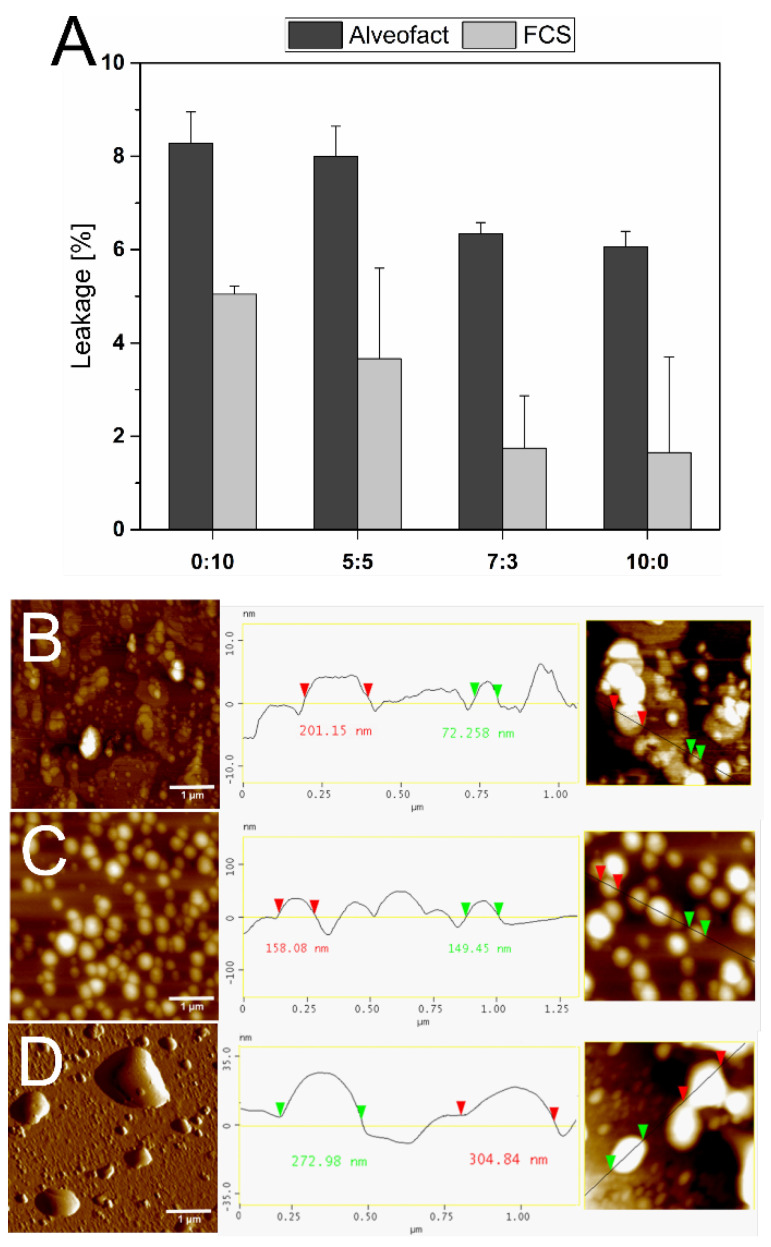
(**A**) The leakage of 5(6)-carboxyfluorescein from liposomes composed of tetraether lipids (TEL) and Phospholipon 100H (Ph) in different molar ratios (0:10, 5:5, 7:3, 10:0; TEL:Ph) after incubation for 4 h with Alveofact and fetal calf serum (FCS), respectively. AFM images displayed in height or phase mode of (**B**) 0:10 TEL:Ph liposomes after incubation for 4 h with FCS, (**C**) 7:3 TEL:Ph liposomes after incubation for 4 h with Alveofact, and (**D**) 0:10 TEL:Ph liposomes after incubation for 4 h with Alveofact. Scale bars represent 1 µm.

All liposome formulations were investigated in terms of stability against Alveofact and FCS. EE of CF was measured after liposome production as previously described. As seen in Table 4, the liposome’s initial EE was approximately 20% in all formulations. Figure 5 illustrates the leakage of CF after 4 h incubation with Alveofact and FCS. With an increasing ratio of TEL in the liposome formulations, the integrity of the liposome structure was preserved. After 4 h incubation, the leakage of CF was below 10% for all formulations. However, liposomes without TEL were the least stable of all tested formulations. 8.28% CF during Alveofact incubation and 5.05% CF during FCS incubation leaked from the liposomes.

Additionally, AFM images in Figure 5 show liposomes before and after 4 h incubation in FCS (0:10 TEL:Ph) and Alveofact (7:3 TEL:Ph, 0:10 TEL:Ph). The 7:3 TEL:Ph liposomes in Figure 5C appear spherical, intact, and resistant against Alveofact. Only a slight increase in diameter to 155.67 nm was recorded (see Table 5). The PDI also showed a monodisperse particle size distribution. Identical results were also observed with FCS for the TEL-containing liposomes (5:5 TEL:Ph, 0:10 TEL:Ph). Ether liposomes, unlike ester liposomes, have resistance against high-density lipoproteins in blood, plasma, or serum because of their bilayer-spanning TEL [52,59]. In terms of pulmonary delivery, this bilayer-spanning property of TEL may also explain the resistance against Alveofact. Ph liposomes tend to aggregate or form larger liposomes when interacting with FCS or Alveofact (Figure 5B,D). The particle size increases significantly to 187.25 nm (FCS) or 252.56 nm (Alveofact). The significantly increased PDI indicates a polydisperse particle size distribution. Interestingly, this morphological change in the shape of the liposomes hardly affected the CF leakage. However, the 10:0 TEL:Ph formulation shows the highest stability.

The TEL proportion seemed to have a particular stabilizing effect under serum and lung surfactant incubation. Patel et al. pointed out that leakage of CF and fluorescein-conjugated bovine serum albumin occurred in TEL liposomes during the incubation in simulated human bile because of vesicle disruption rather than increased permeability [60]. In contrast, the AFM image of 7:3 TEL:Ph liposomes show well-shaped particles after incubation for 4 h with FCS (Figure 5C). The CF leakage results presented in Figure 5 agree with Choquet et al. [52]. Increasing amounts of TEL in the lipid bilayer reduced leakage of CF in the presence of both Alveofact and FCS. The positive impact on retention may be advantageous in pulmonary and oral drug delivery since the conditions inside the lung and the gastrointestinal tract might induce degradation of the leaked drugs.

## 4. Conclusions

In this study, the stability properties of liposomes have been addressed in terms of their potential applications in lung and gastrointestinal environments. It was demonstrated that TELs enhance the stability of liposomes, primarily if they represent more than 50% of the formulation. Since the TELs are adapted to very high temperatures, sterilizing liposomes by autoclavation is possible and provides considerable advantages for clinical applications. This approach proves that the TEL molar ratio has a distinctive effect on the stability with only slight variations in size and PDI against various pH levels, temperature conditions, and biological environments (FCS and surfactant). Thus, these liposomes offer attractive possibilities for treatment applications aimed at the pulmonary system and the gastrointestinal tract.

## Figures and Tables

**Table 1:** The mean hydrodynamic diameter, polydispersity index (PDI), zeta potential, and encapsulation efficiency (EE) of liposomes composed of tetraether lipids (TEL) and Phospholipon 100H (Ph) in different molar ratios (0:10, 5:5, 7:3, 10:0; TEL:Ph).

Formulation	Hydrodynamic Diameter [nm]	PDI	Zeta Potential [mV]
0:10 TEL:Phospholipon 100H	34.56 ± 0.77	0.24 ± 0.01	−19.40 ± 0.03
5:5 TEL:Phospholipon 100H	99.64 ± 1.04	0.19 ± 0.02	−42.50 ± 1.41
7:3 TEL:Phospholipon 100H	107.00 ± 0.53	0.12 ± 0.02	−45.50 ± 0.63
10:0 TEL:Phospholipon 100H	135.60 ± 0.03	0.16 ± 0.03	−49.90 ± 0.91

**Table 2:** The polydispersity index (PDI) of liposomes composed of tetraether lipids (TEL) and Phospholipon 100H (Ph) in different molar ratios (0:10, 5:5, 7:3, 10:0; TEL:Ph) incubated for 4 h at 25, 36, and 60 °C.

Formulation	PDI		
	25 °C	36 °C	60 °C
0:10 TEL:Phospholipon 100H	0.24 ± 0.04	0.25 ± 0.08	0.44 ± 0.11
5:5 TEL:Phospholipon 100H	0.16 ± 0.01	0.15 ± 0.06	0.18 ± 0.01
7:3 TEL:Phospholipon 100H	0.12 ± 0.02	0.13 ± 0.01	0.14 ± 0.02
10:0 TEL:Phospholipon 100H	0.15 ± 0.03	0.17 ± 0.04	0.17 ± 0.05

**Table 3:** The polydispersity index (PDI) of liposomes composed of tetraether lipids (TEL) and Phospholipon 100H (Ph) in different molar ratios (0:10, 5:5, 7:3, 10:0; TEL:Ph) incubated for 4 d at pH 2.0, 4.0, 7.4, and 9.0, respectively.

Formulation	PDI			
	pH 2.0	pH 4.0	pH 7.4	pH 9.0
0:10 TEL:Phospholipon 100H	0.43 ± 0.02	0.31 ± 0.05	0.20 ± 0.11	0.28 ± 0.04
5:5 TEL:Phospholipon 100H	0.22 ± 0.09	0.23 ± 0.02	0.12 ± 0.02	0.14 ± 0.02
7:3 TEL:Phospholipon 100H	0.06 ± 0.05	0.12 ± 0.02	0.13 ± 0.02	0.15 ± 0.07
10:0 TEL:Phospholipon 100H	0.14 ± 0.02	0.15 ± 0.03	0.14 ± 0.04	0.24 ± 0.03

**Table 4:** The encapsulation efficiency (EE) of liposomes composed of tetraether lipids (TEL) and Phospholipon 100H (Ph) in different molar ratios (0:10, 5:5, 7:3, 10:0; TEL:Ph).

Formulation	Encapsulation Efficiency (EE)
0:10 TEL:Phospholipon 100H	18.80 ± 3.81%
5:5 TEL:Phospholipon 100H	22.73 ± 0.21%
7:3 TEL:Phospholipon 100H	20.43 ± 5.71%
10:0 TEL:Phospholipon 100H	20.48 ± 4.94%

**Table 5:** The mean hydrodynamic diameter and polydispersity index (PDI) of liposomes composed of tetraether lipids (TEL) and Phospholipon 100H (Ph) in different molar ratios (0:10, 5:5, 7:3, 10:0; TEL:Ph) after 4 h incubation with Alveofact and fetal calf serum (FCS), respectively.

Formulation	FCS		Alveofact	
	Diameter [nm]	PDI	Diameter [nm]	PDI
0:10 TEL:Phospholipon 100H	187.25 ± 7.95	0.38 ± 0.06	252.56 ± 6.88	0.44 ± 0.07
5:5 TEL:Phospholipon 100H	132.21 ± 2.38	0.17 ± 0.04	151.71 ± 3.22	0.22 ± 0.03
7:3 TEL:Phospholipon 100H	148.65 ± 3.31	0.19 ± 0.02	155.67 ± 3.45	0.20 ± 0.04
10:0 TEL:Phospholipon 100H	145.70 ± 0.03	0.19 ± 0.03	142.35 ± 2.64	0.18 ± 0.04

## Data Availability

This publication is based upon the author’s thesis (Aybike Özcetin now named Aybike Hemetsberger, University of Marburg, 2011, DOI: 10.17192/z2011.0109), titled “Tetraether lipid Liposomes for the Preparation of Novel Liposomal Drug Carriers”.

## Supplementary Materials

The following supporting information can be downloaded at: https://www.mdpi.com/article/10.3390/ma15196995/s1, Figure S1: Limulus amebocyte lysate test of Control Standard Endotoxin as positive control, pyrogen-free water as negative control, and 10:0 TEL: Phospholipon 100H liposomes.
