# Supplementary material for: Highly Stable Liposomes Based on Tetraether Lipids as a Promising and Versatile Drug Delivery System
Source: Materials (Basel). 2022 Oct 9;15(19):6995. doi: 10.3390/ma15196995 (PMC9571198; doi:10.3390/ma15196995)
Supplement: Supplementary file 1 [file materials-15-06995-s001.zip › materials-1916381-supplementary.pdf]

materials

Supplementary material

Highly Stable Liposomes Based on Tetraether Lipids as a Promising and Versatile Drug Delivery System

Aybike Hemetsberger [1,†], Eduard Preis [1,†], Konrad Engelhardt [1], Bernd Gutberlet [1], Frank Runkel [2,3] and Udo Bakowsky [1,*]

[1] Department of Pharmaceutics and Biopharmaceutics, University of Marburg, Robert-Koch-Str. 4, 35037 Marburg, Germany
[2] Institute of Bioprocess Engineering and Pharmaceutical Technology, University of Applied Sciences Mittelhessen, Wiesenstrasse 14, 35390 Giessen, Germany
[3] Faculty of Biology and Chemistry, Justus-Liebig University Giessen, Heinrich-Buff-Ring 17, 35392 Giessen, Germany
* Correspondence: ubakowsky@aol.com
† These authors contributed equally to this work.

Materials and methods

Limulus amebocyte lysate test

Control Standard Endotoxin (CSE, *Escherichia coli* O113:H10, 0.5µg/vial) (Associates of Cape Cod Europe GmbH, Mörfelden-Walldorf, Germany) and limulus amebocyte lysate (LAL, sensitivity: 0.125 EU/mL) (Associates of Cape Cod Europe GmbH, Mörfelden-Walldorf, Germany) were prepared according to the manufacturer's instructions. 100 µL 10:0 TEL:Ph liposomes, pyrogen-free water as negative control, and CSE as positive control were transferred to depyrogenated reaction tubes. 100 µL LAL solution were added to each reaction tube and carefully mixed. These samples were incubated for 60 min at 37 °C. Then the tubes were carefully taken out with a test tube forceps and rotated 180°. The results were recorded by a camera.

Results and discussion

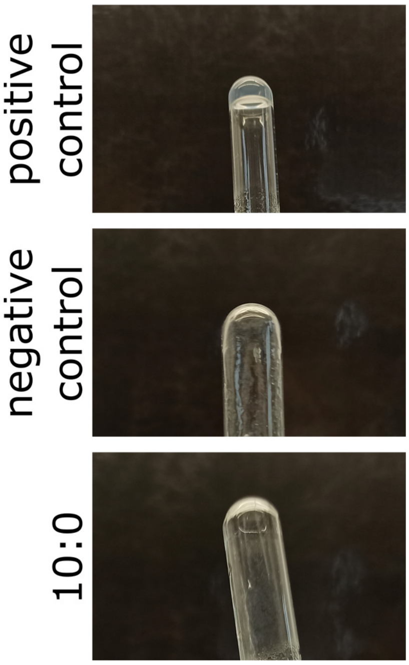

Figure S1. Limulus amebocyte lysate test of Control Standard Endotoxin as positive control, pyrogen-free water as negative control, and 10:0 TEL:Phospholipon 100H liposomes.
